# Supplementary material for: A Single Dose of a Hybrid hAdV5-Based Anti-COVID-19 Vaccine Induces a Long-Lasting Immune Response and Broad Coverage against VOC
Source: Vaccines (Basel). 2021 Sep 29;9(10):1106. doi: 10.3390/vaccines9101106 (PMC8537385; doi:10.3390/vaccines9101106)
Supplement: Supplementary file 1 [file vaccines-09-01106-s001.zip › vaccines-1368068-SM.pdf]

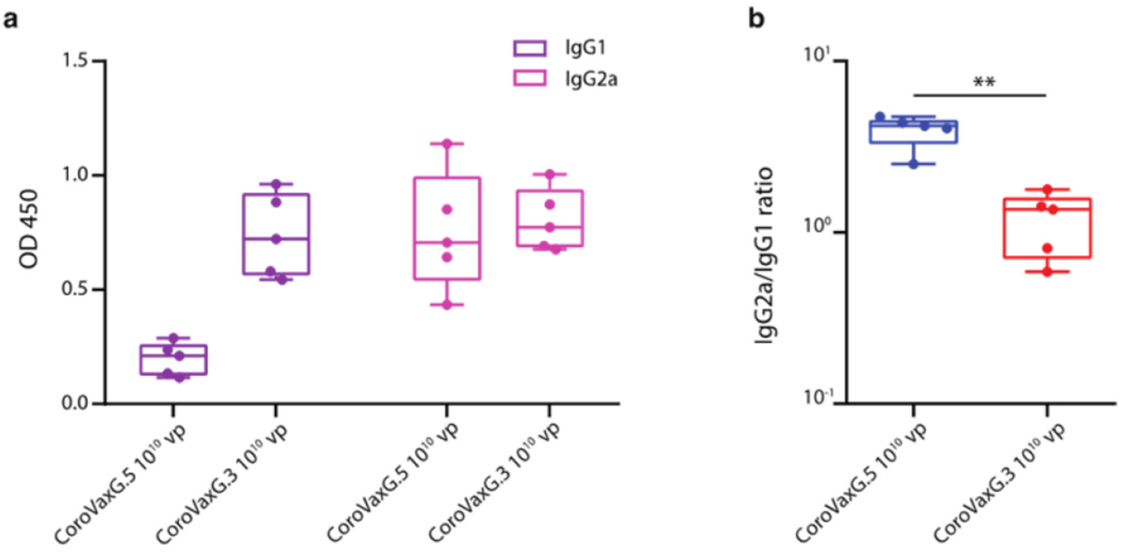

Figure S1. Anti-Spike IgG subclass reactivity at 14 days post vaccination. Six-week-old BALB/c mice (n = 5/group) received immunizations with 1010 vp of an Ad-vectored vaccine. Sera were collected at 14 days post vaccination and IgG1 and IgG2a anti-S was detected by ELISA. (a) Optical density at 450 nm determined by ELISA of a 1:50 dilution of the sera. (b) IgG2a/IgG1 ratio. The box plots show median, 25th and 75th percentiles and the whiskers show the range. **P < 0.01; two-tailed Mann-Whitney U test.
